# Characterization of Ruddlesden-Popper La_2−*x*_Ba*_x_*NiO_4±δ_ Nickelates as Potential Electrocatalysts for Solid Oxide Cells

**DOI:** 10.3390/ma16041755

**Published:** 2023-02-20

**Authors:** Kiryl Zakharchuk, Andrei Kovalevsky, Aleksey Yaremchenko

**Affiliations:** Department of Materials and Ceramic Engineering, CICECO—Aveiro Institute of Materials, University of Aveiro, 3810-193 Aveiro, Portugal

**Keywords:** nickelate, electrical conductivity, ionic conductivity, thermal expansion, electrocatalyst, NO*_x_* elimination

## Abstract

Ruddlesden-Popper La_2−*x*_Ba*_x_*NiO_4±δ_ (*x* = 0–1.1) nickelates were prepared by a glycine-nitrate combustion route combined with high-temperature processing and evaluated for potential application as electrocatalysts for solid oxide cells and electrochemical NO*_x_* elimination. The characterization included structural, microstructural and dilatometric studies, determination of oxygen nonstoichiometry, measurements of electrical conductivity and oxygen permeability, and assessment of chemical compatibility with other materials. The formation range of phase-pure solid solutions was found to be limited to *x* = 0.5. Exceeding this limit leads to the co-existence of the main nickelate phase with low-melting Ba- and Ni-based secondary phases responsible for a strong reactivity with Pt components in experimental cells. Acceptor-type substitution of lanthanum by barium in La_2−*x*_Ba*_x_*NiO_4+δ_ is charge-compensated by decreasing oxygen excess, from δ ≈ 0.1 for *x* = 0 to nearly oxygen-stoichiometric state for *x* = 0.5 at 800 °C in air, and generation of electron-holes (formation of Ni^3+^). This leads to an increase in *p*-type electronic conductivity (up to ~80 S/cm for highly porous La_1.5_Ba_0.5_NiO_4+δ_ ceramics at 450–900 °C) and a decline of oxygen-ionic transport. La_2−*x*_Ba*_x_*NiO_4+δ_ (*x* = 0–0.5) ceramics exhibit moderate thermal expansion coefficients, 13.8–14.3 ppm/K at 25–1000 °C in air. These ceramic materials react with yttria-stabilized zirconia at 700 °C with the formation of an insulating La_2_Zr_2_O_7_ phase but show good chemical compatibility with BaZr_0.85_Y_0.15_O_3−δ_ solid electrolyte.

## 1. Introduction

The decomposition of hazardous NO*_x_* gases, which are formed under lean combustion conditions (mostly NO (90–95%) and NO_2_ (5–10%)), remains a significant problem due to the passivation of traditional three-way catalysts by oxygen. In the case of stoichiometric combustion conditions, the composition of exhaust gases containing unreacted and hazardous substances can be brought to thermodynamic equilibrium by a catalyst, with the formation of H_2_O, CO_2_, and N_2_. The presence of oxygen in exhaust gases makes it difficult to reduce NO*_x_* to nitrogen; reducing agents, CO, and unburned hydrocarbons react predominantly with oxygen, and the active sites of catalysts are occupied by oxygen species. Direct thermal decomposition of NO is thermodynamically favorable but limited by kinetics and high activation energy of decomposition [1,2].

There are several approaches to reduce nitrogen oxide in the presence of oxygen in exhaust gases. Selective Catalytic Reduction systems using ammonia and urea as reductants are commercialized and widely used for stationary applications and heavy-duty vehicles. Implementing these systems in light vehicles and domestic gas-based water heaters introduces complexity and excessive cost. A promising alternative way to avoid additional chemicals is a combination of NO*_x_* storage with electrochemical reduction using a solid oxide electrolyte cell. In the 1970s, it was shown that a solid oxide cell with Pt electrodes and yttria-stabilized zirconia (YSZ) membrane as an electrolyte is capable of reducing NO to nitrogen with a high conversion rate [3,4]. Cathodic polarization makes it possible to overcome the potential barrier of NO decomposition and refresh surface oxygen vacancies acting as catalytically active sites. However, passivation by oxygen remained relevant for electrochemical cells due to competition between NO and O_2_ reduction. Conversion efficiency has been improved in more recent studies by replacing the Pt electrodes with oxide mixed ionic-electronic conductors (MIECs), impregnating electrodes with BaO/K_2_O storage materials, and pre-oxidizing NO to NO_2_ on additional catalyst [5,6,7,8]. Pre-oxidation is a key factor as NO_2_ is readily absorbed by storage material and, consequently, NO*_x_* selectivity increases. Recent research on electrochemical NO*_x_* reduction focuses on developing selective electrode materials able to oxidize/store/reduce NO_x_ in the presence of up to 15 vol.% of oxygen.

Potentially, Ba-containing MIEC oxides may perform the function of oxidation/storage/reduction during redox cycles in a solid oxide cell without impregnation with storage materials and the use of an additional oxidation catalyst. The catalytic activity of transition-metal-based LaMO_3_ perovskites to NO*_x_* decomposition is known to increase in the order M = Cr < Fe < Mn < Co < Ni [9,10]. Taking this into account, perovskite-related La_2−*x*_Ba*_x_*NiO_4±δ_ nickelates with layered Ruddlesden-Popper structure seem to be a promising choice for electrochemical reduction application. These ceramic materials impregnated with BaCO_3_/BaO were demonstrated to exhibit catalytic activity for direct thermal decomposition of NO even in the presence of 2% of oxygen [11]. The highest conversion efficiency of a gas mixture containing 4% NO and 2% O_2_ was found for La_1.2_Ba_0.8_NiO_4±δ_. The origin of catalytic activity was attributed to the formation of oxygen vacancies and Ni^2+/3+^ redox couples induced by acceptor-type substitution of lanthanum by barium. Both factors are assumed to be responsible for lowering the activation energy of decomposition by the formation of intermediate complexes, in addition to the basic nature of Ba-containing ceramics which helps to adsorb acidic NO*_x_* gases [11,12].

La_2_NiO_4+δ_ (where δ is oxygen nonstoichiometry) and its derivatives are attractive MIEC electrode materials for solid oxide fuel and electrolysis cells due to a favorable combination of properties including substantial electronic conductivity, high oxygen diffusivity, suitable thermal expansion, and phase stability in a wide range of T-p(O_2_) conditions [13,14,15,16]. Acceptor-type substitutions of lanthanum by strontium or barium lead to gradual changes in defect chemistry, level of electrical conductivity, and mechanism of oxygen diffusion in the perovskite-related lattice [16,17,18,19,20,21,22,23]. The formation range of single-phase La_2−*x*_Ba*_x_*NiO_4±δ_ solid solutions varies in different literature reports: up to *x* = 0.7 [24] and *x* = 1.0 [22] for the series prepared by solid-state route in air, and *x* = 1.1 for the materials obtained by co-precipitation method [25] and Pechini-type route [23]. The presence of amorphous phase impurities mentioned in [26] may be one of the reasons responsible for the discrepancy in the literature. At the same time, the presence of Ba-rich impurities such as BaO or BaCO_3_ may be favorable for application in electrocatalytic NO_x_ decomposition [11,27], as these basic phases act as storage materials and promote the absorption of acidic NO*_x_* gases. 

The present work is focused on the preparation and characterization of La_2−*x*_Ba*_x_*NiO_4±δ_ (*x* = 0–1.1) nickelates for potential application as electrocatalysts for electrochemical NO*_x_* elimination. Particular attention was given to synthetic and processing procedures, solid solution formation range, oxygen nonstoichiometry, electrical transport properties, and thermomechanical and chemical compatibility with other components of solid electrolyte cells.

## 2. Materials and Methods

Synthesis of La_2−*x*_Ba*_x_*NiO_4±δ_ (*x* = 0–1.1) nickelates was performed by glycine-nitrate combustion technique. La_2_O_3_ (Sigma-Aldrich, St. Louis, MO, USA, 99.99%), BaCO_3_ (Sigma-Aldrich, 99+%) and NiO (Alfa Aesar, 99%) were used as starting precursors. Before weighing, lanthanum oxide was calcined at 1000 °C for 2 h to decompose adsorbates. The precursors were dissolved in a minimum required amount of diluted nitric acid to yield aqueous nitrate solutions containing metal cations in appropriate proportions. Glycine (Sigma-Aldrich, ≥99%) was added into the solutions with glycine/nitrate molar ratio double of stoichiometric (assuming H_2_O, CO_2_ and N_2_ to be the only gaseous products of redox reaction). After stirring for several hours, each solution was heated on a hot plate until evaporation of water and auto-ignition. The ash-like products of combustion were calcined in air at 800 °C to burn out organic residues. This was followed by repeated calcinations of powders with a stepwise (50–100 °C) increase in calcination temperature and intermediate regrindings: at 900–1000 °C in air for *x* = 0–0.5 and 900–1100 °C in O_2_ for *x* = 0.6–1.1. The adoption of a multi-stage calcination route after the combustion process was necessary to promote homogenization and formation of the target phase in the purest possible state, which is hampered due to the existence of numerous phases in the La-Ni-O and Ba-Ni-O systems as well as due to the prone nature of lanthanum and barium precursors to form hydroxides and carbonates in ambient air. As an example, Figure S1 (see Supplementary Materials) illustrates the evolution of the X-ray diffraction (XRD) patterns for one of the compositions in the course of the synthetic process. Ceramics samples for dilatometric studies and measurements of electrical transport properties were prepared by uniaxial compaction of the powders into disk-shaped pellets followed by isostatic pressing at 200 MPa and sintering at 1100–1350 °C for 5 h in air (*x* ≤ 0.5) or flowing oxygen (*x* ≥ 0.6). Sintering conditions (temperature and atmosphere) were selected for each composition individually based on preliminary results and suggestions in the literature [17,23,25].

Experimental density was calculated from the mass and dimensions of ceramic samples polished after sintering using SiC grinding paper. Bar-shaped samples (approximate dimensions 1.5 mm × 2.5 mm × 13 mm) for dilatometric and electrical measurements were cut out of sintered pellets using a Struers Minitom precision cutting machine with a diamond cut-off wheel. Powdered samples for XRD studies and thermogravimetric analysis were prepared by grinding sintered ceramics in a mortar. 

Room-temperature XRD patterns were recorded using PANalytical X’Pert PRO (PANalytical, Almelo, The Netherlands, CuK_α_ radiation, step 0.026°) and Rigaku D/Max-B (Rigaku, Tokyo, Japan, CuK_α_ radiation, step 0.02°) diffractometers in the range 2θ = 20–80° for phase analysis and 20–120° for calculations of the lattice parameters. The lattice parameters were calculated in FullProf software (version March 2021). In order to assess possible changes in the phase composition on thermal cycling, variable-temperature XRD data were collected using a PANalytical X’Pert PRO diffractometer equipped with an Anton Paar HTK 16N high-temperature chamber and with Pt foil used as a sample support; the patterns were recorded on heating in air in the temperature range 25–1300 °C with the step of 100 °C and equilibration for 10 min at each step before the data acquisition. Scanning electron microscopy (SEM, Hitachi SU-70 microscope, Hitachi, Tokyo, Japan) coupled with energy dispersive spectroscopy (EDS, Bruker Quantax 400 EDS detector, Bruker, Berlin, Germany) was used for microstructural characterization and detection of secondary phases.

**Figure 1:**
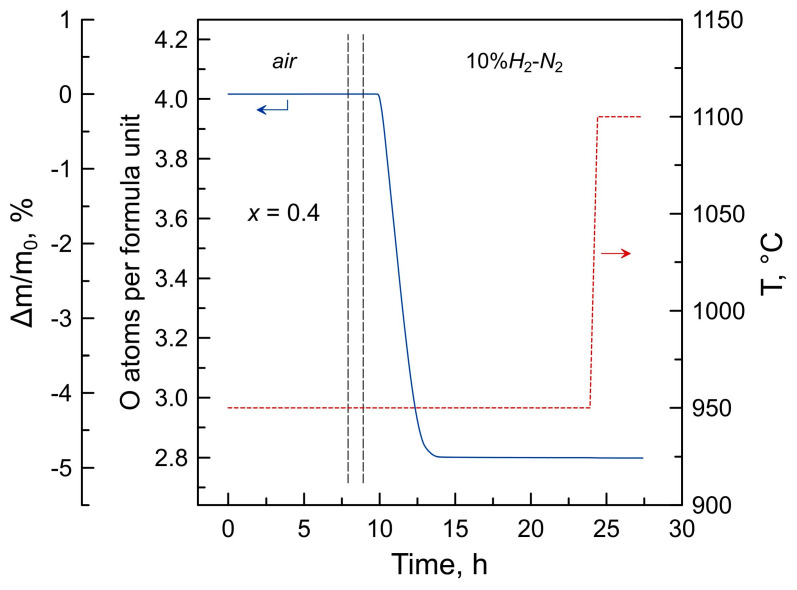
Relative weight changes and corresponding changes in the oxygen content per La_2−*x*_Ba*_x_*NiO_4+δ_ formula unit as a function of time. The plot illustrates the determination of the absolute oxygen nonstoichiometry δ of La_1.6_Ba_0.4_NiO_4+δ_ in the reference state (air, 950 °C) via isothermal reduction to a mixture of Ni with lanthanum and barium oxides in the 10% H_2_-N_2_ flow. The system was flushed with Ar for 1 h (marked by vertical dotted lines) between air and reducing atmosphere.

Thermogravimetric analysis (TGA, Setaram SetSys 16/18 instrument, Setaram, Caluire, France, sensitivity 0.4 μg, initial sample weight 0.5–1.0 g) was carried out in flowing air on heating/cooling at 25–1000 °C with a constant rate of 2 °C/min or with isothermal equilibration steps (3 h) at 700–950 °C. After thermal cycling in air, each sample was reduced isothermally at 950 °C in flowing 10% H_2_-N_2_ flow (Figure 1) in order to determine the absolute oxygen content. The reduction step was 15 h long to ensure the complete transformation into a mixture of metallic nickel with lanthanum and barium oxides (Figure S2). All thermogravimetric data were corrected for buoyancy effects by subtracting the corresponding baselines recorded under identical conditions using a dense inert ceramic sample of a similar volume. Dilatometric studies were performed employing a vertical Linseis L75 dilatometer in flowing air in the temperature range between room temperature and 1000–1100 °C with a constant heating/cooling rate of 3 °C/min. 

The electrical conductivity (σ) of ceramic samples was determined by the 4-probe DC (direct current) method using bar-shaped samples and Pt wires as probes and current collectors. The end-facing surfaces of the bars were covered with Pt paint (Heraeus CL11-5349) to improve electrical contact. The measurements were performed as a function of temperature in air and isothermally as a function of oxygen partial pressure in flowing N_2_-O_2_ mixtures with equilibration at each T-p(O_2_) data point. The gas flow rates were set by Bronkhorst mass-flow controllers. The measurements of oxygen permeation fluxes through dense ceramic membranes were performed at 700–950 °C using electrochemical YSZ solid electrolyte cells comprising an oxygen pump and a sensor [28,29]. The oxygen partial pressure at the membrane feed side (*p*_2_) was equal to 0.21 atm (atmospheric air). The gas-tightness of the membranes before the measurements was verified by the absence of physical leakage under the total pressure gradient of 2–3 atm at room temperature.

In all experiments, air was supplied by Jun-Air air compressors equipped with a drying unit; the relative humidity in the supplied air flow was ≈10% at room temperature. Oxygen partial pressure during the experiments in controlled atmospheres was monitored using electrochemical YSZ sensors.

To assess the high-temperature chemical compatibility of lanthanum-barium nickelates with other potential components of electrochemical cells, the mixtures of selected La_2−*x*_Ba*_x_*NiO_4±δ_ samples with (ZrO_2_)_0.92_(Y_2_O_3_)_0.08_ (8YSZ, Tosoh, Tokyo, Japan), BaZr_0.85_Y_0.15_O_3−δ_ (BZY15, CerPoTech, Tiller, Norway), Pt and Au powders were annealed at 700 °C for 72 h and examined by XRD. Platinum and gold powders were prepared by dissolving pieces of metallic wires in aqua regia followed by drying and thermal decomposition of obtained chloroplatinic and chloroauric acids in the flow of 10% H_2_-N_2_ at 300 °C. Dense ceramic samples of 8YSZ and BZY15 for dilatometric measurements were sintered at 1600 °C/10 h and 1670 °C/10 h, respectively. During sintering, BZY15 compacts were covered with a thick layer of the BZY15 powder to avoid the evaporation of barium.

## 3. Results

### 3.1. Phase Composition, Crystal Structure and Microstructure

**Figure 2:**
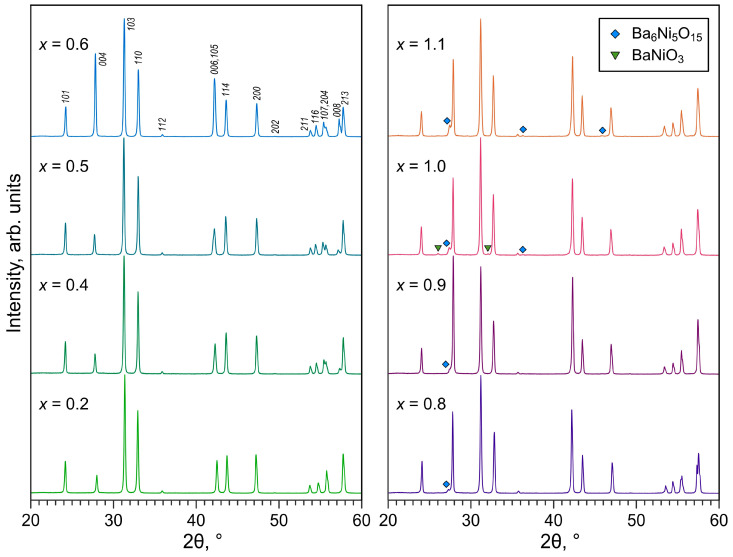
Room-temperature XRD patterns of La_2−*x*_Ba*_x_*NiO_4±δ_ ceramics sintered at 1200 °C in air (*x* ≤ 0.5) or in oxygen (*x* ≥ 0.6). After sintering, the powdered samples were annealed in air at 900 °C for 2 h and slowly cooled. The reflections of the main phase are indexed in the *I*4/*mmm* space group. The reflections of secondary phases are marked according to ICDD PDF 04-007-8462 (hexagonal BaNiO_3_) and 04-009-3992 (rhombohedral Ba_6_Ni_5_O_15_).

X-ray diffraction of sintered La_2−*x*_Ba*_x_*NiO_4±δ_ ceramic samples showed the formation of solid solutions with Ruddlesden–Popper K_2_NiF_4_-type structures for the entire range of prepared compositions (Figure 2). The compositions with moderate barium contents, *x* ≤ 0.6, were virtually phase-pure. XRD patterns of the Ba-rich (*x* ≥ 0.8) samples exhibited additional reflections assigned to the secondary phases of the BaO-NiO system; the amount of impurity phases increased with increasing nominal barium content (Figure 2). Preferred orientation effects indicated by the elevated intensity of (*00l*) reflections can also be observed in the XRD patterns of the *x* = 0.6–1.1 samples, which is rather common for Ruddlesden–Popper layered structures due to the anisotropy of ceramic fracture during milling.

**Figure 3:**
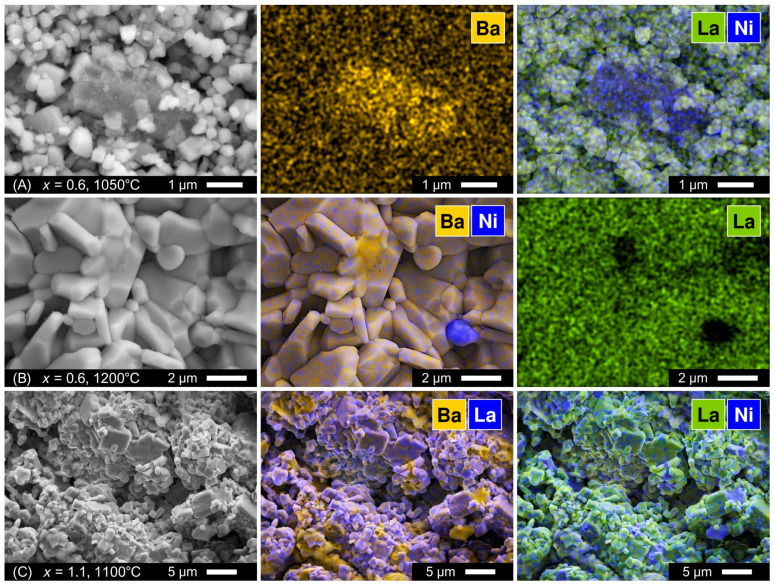
SEM micrographs of La_2−*x*_Ba*_x_*NiO_4±δ_ samples and corresponding EDS elemental mapping: (**A**) as-prepared La_1.4_Ba_0.6_NiO_4±δ_ powder; (**B**) fractured cross-section of La_1.4_Ba_0.6_NiO_4±δ_ ceramics sintered at 1200 °C; (**C**) fractured cross-section of La_0.9_Ba_1.1_NiO_4±δ_ ceramics sintered at 1100 °C.

One should note that X-ray diffraction is often not sensitive to small fractions of impurities (≤3–4 wt.%) as well as to amorphous precipitates. While the XRD patterns of La_1.4_Ba_0.6_NiO_4±δ_ samples showed no evidence of secondary phases (Figure 2), SEM combined with EDS elemental mapping revealed the presence of Ba-Ni-O phase inclusions, Ba-rich precipitates (oxide or carbonate), and NiO particles in both as-synthesized powder and sintered ceramics (Figure 3A,B). The size of the Ba-Ni-O inclusions in the powder was typically several times larger compared to the regular particles of the La_2_NiO_4_-based phase (0.3–1.0 µm, Figure 3A). The inclusions are likely agglomerates of grains of the main phase bound together by the amorphous Ba-rich phase. 

Combined XRD and SEM/EDS results imply that the formation of single-phase La_2−*x*_Ba*_x_*NiO_4±δ_ solid solutions is restricted to the 0 ≤ *x* ≤ 0.5 range, at least under processing conditions employed in the present work. Tang et al. [25] discussed the comparatively limited solubility of barium cation in the lanthanum sublattice of La_2_NiO_4±δ_ in terms of the Goldschmidt tolerance factor. The Goldschmidt tolerance factor *t* is a dimensionless parameter describing the bond length matching between different layers of the crystal lattice and is used as an indicator for the stability or distortions of selected crystal structures. For the perovskite-related RP-type A_2_BO_4_ structure, the tolerance factor is given by:

(1) $$t=\frac{r_{A}^{\mathrm{IX}}+r_{O}}{\sqrt{2}(r_{B}^{\mathrm{VI}}+r_{O})}$$

**Figure 4:**
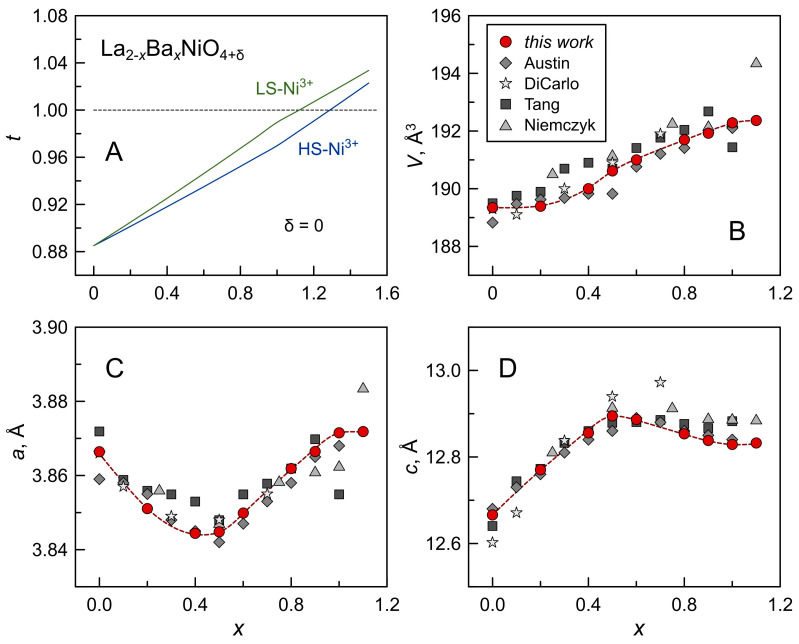
(**A**) Variation of Goldshmidt tolerance factor for the La_2−*x*_Ba*_x_*NiO_4_ series with barium content (estimated assuming δ = 0 and using ionic radii from Shannon [30]; HS and LS indicate high-spin and low-spin Ni^3+^, respectively). (**B**–**D**) Unit cell parameters of tetragonal lattice (space group *I*4/*mmm*) of sintered La_2−*x*_Ba*_x_*NiO_4±δ_ ceramics. Literature data from Austin [22], DiCarlo [24], Tang [25] and Niemczyk [23] are shown for comparison.

The coordination number of oxygen anions is debatable but often assumed to be VI in perovskite-like structures. Empirically, the tetragonal RP-type A_2_BO_4_ structure is stable over the approximate range of 0.85 < *t* < 1.00 [16]. If the tolerance factor exceeds 1, the A cation is too large (or the B cation is too small) for a tetragonal structure, and the formation of a hexagonal structure is favorable. Figure 4A shows the variation of the tolerance factor for the La_2−*x*_Ba*_x_*NiO_4±δ_ series estimated neglecting oxygen nonstoichiometry and assuming that oxygen ions are in O^2−^ (CN = 6) state and that acceptor-type substitution by Ba^2+^ is charge-compensated by Ni cations in 3+/4+ oxidation states. The simple estimations show that the tetragonal lattice of La_2−*x*_Ba*_x_*NiO_4±δ_ can be expected to form up to *x* = 1.0–1.1 (Figure 4A). Tang et al. [25] argued, however, that a larger thermal expansion of more ionic (La,Ba)-O bonds compared to Ni-O bonds on heating should result in an increasing mismatch in the bond lengths, thus further narrowing the range of barium solid solubility at elevated temperatures.

**Figure 5:**
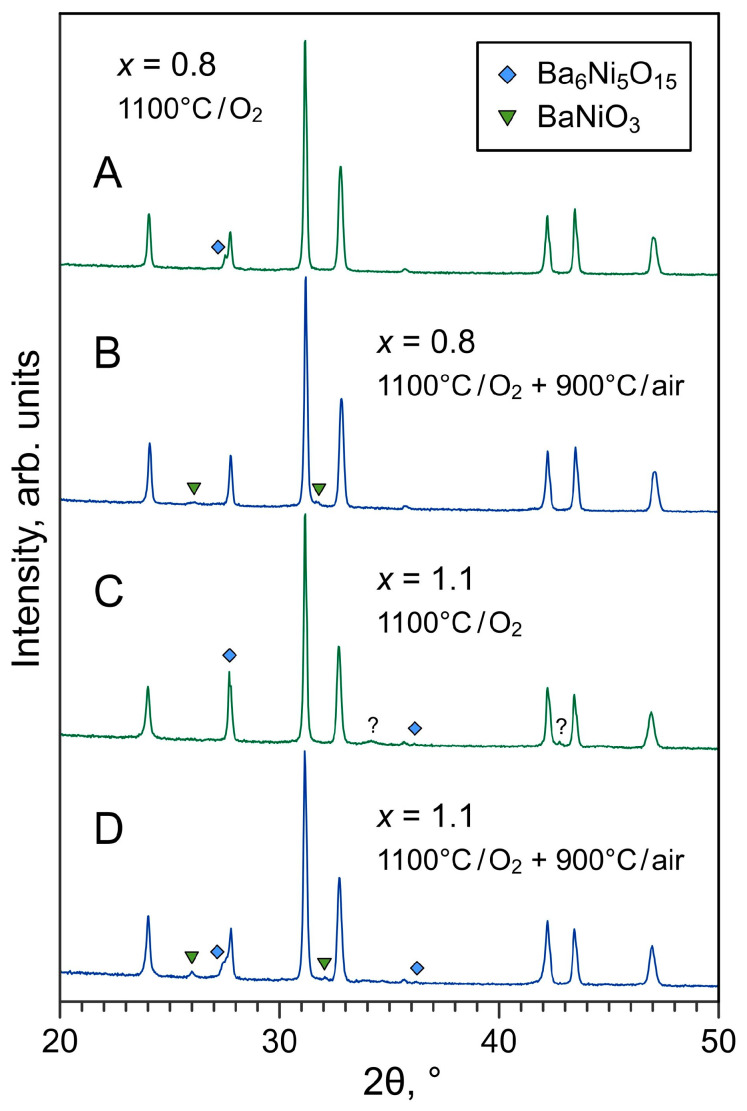
XRD patterns of La_2−*x*_Ba*_x_*NiO_4±δ_ ceramics (*x* = 0.8 and 1.1) sintered at 1100 °C for 5 h in oxygen (**A**,**C**) and subsequently annealed at 900 °C for 2 h in air (**B**,**D**). The reflections of secondary phases are marked according to ICDD PDF 04-007-8462 (hexagonal BaNiO_3_) and 04-009-3992 (rhombohedral Ba_6_Ni_5_O_15_).

The formation range of phase-pure La_2−*x*_Ba*_x_*NiO_4±δ_ solid solutions observed in the present work is narrower compared to previous reports on this system [22,23,24,25], however, it agrees well with the results reported by Takeda et al. who showed that the solubility limit of barium in Nd_2−*x*_Ba*_x_*NiO_4±δ_ corresponds to *x* = 0.6 [31]. Taking into account the arguments on possible variations of Ba solubility with temperature [25] and also somewhat lower processing temperatures used in other works [23,25], attempts were made to reduce the sintering temperature of Ba-rich ceramics down to 1100 °C. However, careful inspection of the XRD data revealed that the samples still comprised phase impurities (Figure 5), although sometimes they are not easy to detect, partly due to small fractions or peaks overlapping. Note that the available phase diagram of the BaO-NiO system shows the existence of low-melting compounds with melting and eutectic points between 1080 and 1240 °C [32,33,34]. It is likely that Ba-Ni-O phase impurities remain in a partially amorphous state after sintering of Ba-rich La_2−*x*_Ba*_x_*NiO_4±δ_ ceramics at 1100–1200 °C but undergo crystallization upon thermal treatment at lower temperatures and become more easily detectable in the XRD patterns (Figure 5B,D). An SEM/EDS inspection of Ba-rich (*x* ≥ 0.8) ceramics samples sintered at 1100 °C confirmed the massive segregation of Ba-Ni-O phase impurities (Figure 3C), in agreement with the XRD data.

The trends in the variation of the calculated room-temperature lattice parameters of La_2−*x*_Ba*_x_*NiO_4±δ_ ceramics (Figure 4B–D) are in good agreement with earlier studies [22,23,24,25] and also resemble the behavior reported for the La_2−*x*_Sr*_x_*NiO_4±δ_ [17,24,35] and Nd_2−*x*_Sr*_x_*NiO_4±δ_ [31,36] series. The unit cell volume generally increases with increasing the nominal barium content, while the *a* and *c* lattice parameters exhibit a non-linear dependence. The parameter *a* decreases with barium content until *x* = 0.4–0.5 and then increases on further substitution; the parameter *c* exhibits an opposite trend and shows a maximum at *x* = 0.5. These dependencies were discussed in earlier works [17,22,35,36,37] in terms of a combined effect of simultaneous changes in several parameters: average ionic radii of A- and B-site cations, electronic configuration of Ni cations and Jahn–Teller distortion of NiO_6_ octahedra, and electrostatic repulsion between A cations along the *c* axis. In particular, the size effects are opposite in two cation sublattices: substitution of lanthanum cations ($r_{{\mathrm{La}}^{3+}}^{\mathrm{IX}}$ = 1.22 Å [30]) by larger barium cations ($r_{{\mathrm{Ba}}^{2+}}^{\mathrm{IX}}$ = 1.47 Å) is accompanied by the oxidation of nickel cations with a corresponding decrease in their size ($r_{{\mathrm{Ni}}^{2+}}^{\mathrm{VI}}$ = 0.69 Å vs. $r_{{\mathrm{Ni}}^{3+}}^{\mathrm{VI}}$ = 0.56–0.60 Å) in order to preserve charge neutrality. Austin et al. [22] suggested that the Jahn–Teller effect and the ionic size effect (larger Ba cations) dominate at lower and higher contents of barium, respectively. 

An important observation is that calculated lattice parameters change smoothly with an increase in the nominal barium content, despite precipitation of secondary phases in Ba-rich ceramics, and these changes agree well with the previous literature reports in which La_2−*x*_Ba*_x_*NiO_4±δ_ solid solutions were discussed as phase-pure (Figure 4B–D). This seems to imply the formation of La_2−*x*_Ba*_x_*NiO_4±δ_ solid solutions up to at least nominal *x* = 1.0 in the present work. However, in the case of Ba-rich compositions (*x* ≥ 0), actual barium content is lower than the nominal, and (La,Ba)_2_NiO_4±δ_ solid solution co-exists with the secondary phases. The deviation from the nominal composition (or relationship between the nominal and actual cation composition of the Ruddlesden-Popper phase) can be given by:

(2) $$La_{2-x}Ba_{x}NiO_{4\pm \delta }\to \left(\frac{2-x}{2-x+a}\right)La_{2-x+a}Ba_{x-a}NiO_{4\pm \delta }+\left(\frac{2a}{2-x+a}\right)\mathrm{BaO}+\left(\frac{a}{2-x+a}\right)\mathrm{NiO}$$

Barium and nickel oxides may exist as individual precipitates, convert to carbonates/hydroxides (in the case of barium), or combine to form low-melting phases of the Ba-Ni-O system [32,33,34], such as hexagonal BaNiO_3_ [38] or rhombohedral Ba_6_Ni_5_O_15_ (BaNi_0.83_O_2.5_) [39].

**Figure 6:**
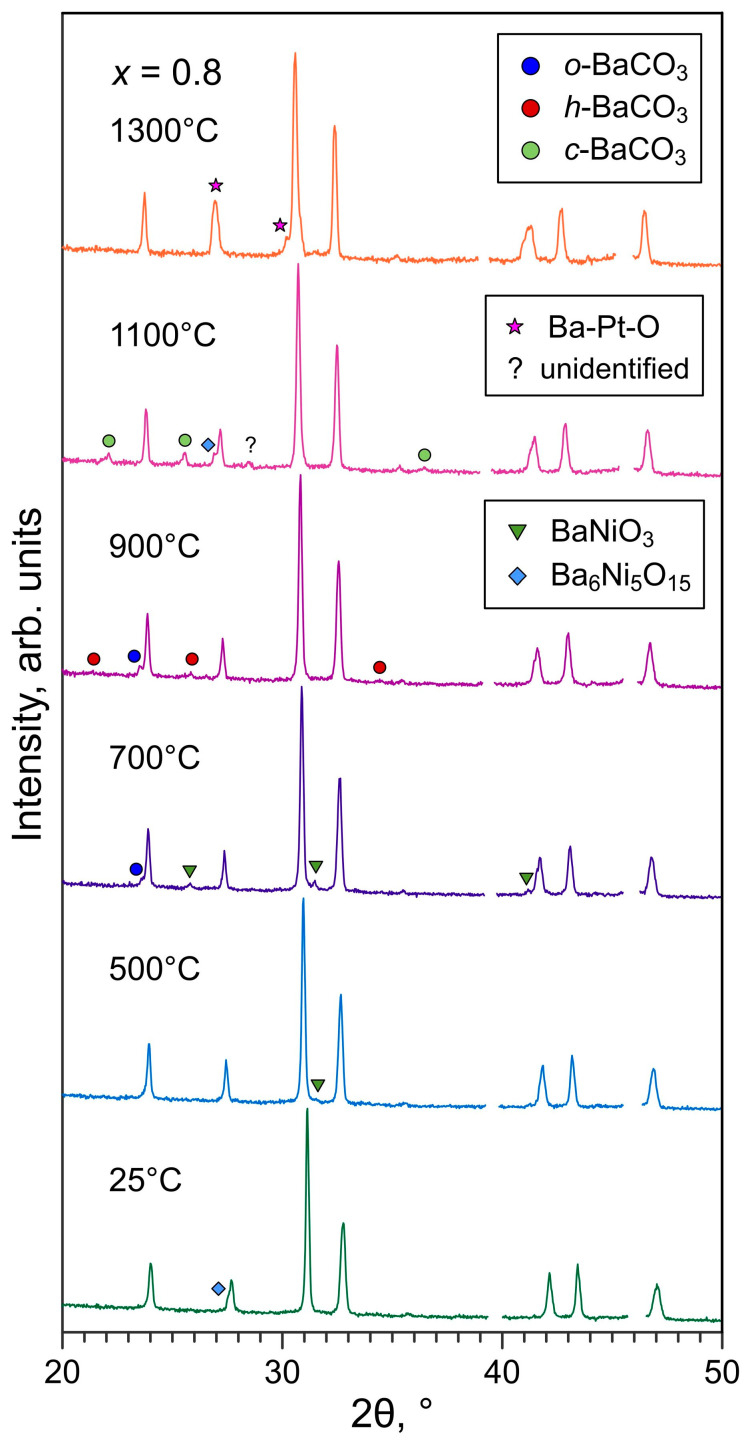
XRD patterns of powdered La_1.2_Ba_0.8_NiO_4±δ_ ceramics (T_sint_ = 1100 °C) recorded on stepwise heating in air. Reflections of Pt foil support are cut out. The reflections of secondary phases are marked according to ICDD PDF 04-007-8462 (hexagonal BaNiO_3_), 04-009-3992 (rhombohedral Ba_6_Ni_5_O_15_), 04-015-3214 (orthorhombic α-BaCO_3_), 04-015-3209 (hexagonal β- BaCO_3_), 00-011-0697 (cubic BaCO_3_), and 04-009-7899 (hexagonal Ba_4_Pt_3_O_9_).

The evolution of the phase composition of Ba-rich ceramics on thermal cycling was studied by variable-temperature XRD for the La_1.2_Ba_0.8_NiO_4±δ_ sample as a representative example. XRD patterns were recorded on stepwise heating with the 100 °C step; selected diffractograms are shown in Figure 6.

The presence of impurity peak ascribed to the Ba_6_Ni_5_O_15_ secondary phase was detected in the initial room-temperature XRD pattern of the as-sintered sample (T_sint_ = 1100 °C). The corresponding peak disappeared on heating to 400 °C. Instead, minor reflections assigned to the BaNiO_3_ phase were evidenced in the diffractogram recorded at 500 °C. The intensity of BaNiO_3_ reflections increased until 700 °C, and these peaks vanished at 800 °C. Instead, traces of the Ba_6_Ni_5_O_15_ phase were detected again at 1100 °C. In parallel, an extra peak assigned to orthorhombic BaCO_3_ was evidenced upon heating to 700 °C, probably due to the crystallization of previously amorphous precipitates. Weak reflections of different polymorphs of barium carbonate could be observed up to 1100 °C. No indication of BaCO_3_ or Ba-Ni-O precipitates could be found in the pattern recorded at 1200 °C, apparently due to the thermal decomposition of carbonate and melting of BaO and Ba-Ni-O phases. Finally, extra peaks originating from a phase of the Ba-Pt-O system (hexagonal Ba_4_Pt_3_O_9_ or Ba_3_Pt_2_O_7_) were detected in the XRD pattern recorded at 1300 °C; the reactivity with platinum is discussed below (Section 3.6). Overall, these observations confirm the precipitation of barium and barium-nickel oxides during the preparation of ceramics at elevated temperatures, with reversible formation/decomposition of barium carbonate and transformations between different Ba-Ni-O phases (for instance, Ref. [40]) on thermal cycling. The presence of BaNiO_3_ (and apparently other secondary phases) was noted earlier in variable-temperature XRD patterns of LaBaNiO_4±δ_ [23].

**Figure 7:**
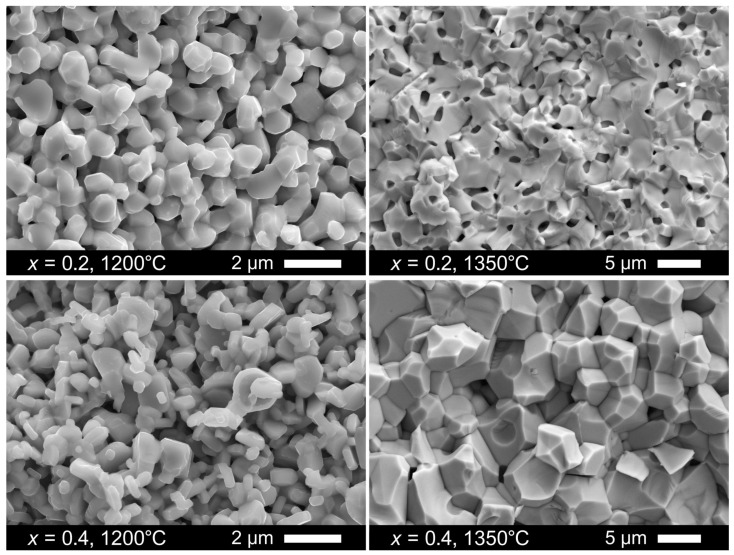
SEM micrographs of fractured cross-sections of La_2−*x*_Ba*_x_*NiO_4+δ_ (*x* = 0.2 and 0.4) ceramics sintered at 1200 and 1350 °C.

The La_2−*x*_Ba*_x_*NiO_4+δ_ nickelates with moderate barium contents, *x* ≤ 0.4, were found to remain stable and phase-pure in air up to at least 1350 °C or even higher (Figure S3) without any microscopic evidence of segregation of secondary phases (Figure S4). Ceramic samples with different porosity (Table 1) were sintered at temperatures between 1100 and 1350 °C. The samples sintered at ≤1200 °C remained highly porous (Figure 7) with an estimated fraction of pores in the range of ~20–40 vol.%. Increasing the sintering temperature to 1350 °C promoted grain growth, from 0.3–1.6 to 3.5–11.0 µm for *x* = 0.4, and densification of ceramics (Figure 7); the relative density reached 92–97% of theoretical. La_2_NiO_4+δ_ and La_1.8_Ba_0.2_NiO_4+δ_ pellets were dense and gas-tight. At the same time, room-temperature tests showed gas leakage through La_1.6_Ba_0.4_NiO_4+δ_ ceramics despite a high relative density (Table 1). RP-type Ln_2_NiO_4+δ_-based materials are known to exhibit an anisotropic thermal expansion of the lattice, with a stronger dilation along the axis *c* [21,41,42,43]. Apparently, grain growth (Figure 7) combined with anisotropic changes in the grain dimensions during thermal cycling promoted intergranular microcracking of La_1.6_Ba_0.4_NiO_4+δ_ ceramics. Earlier, microcracking was reported to be a serious obstacle in the preparation of dense Sr-rich Ln_2−*x*_Sr*_x_*NiO_4−δ_ ceramic materials [21,43].

### 3.2. Oxygen Nonstoichiometry

**Figure 8:**
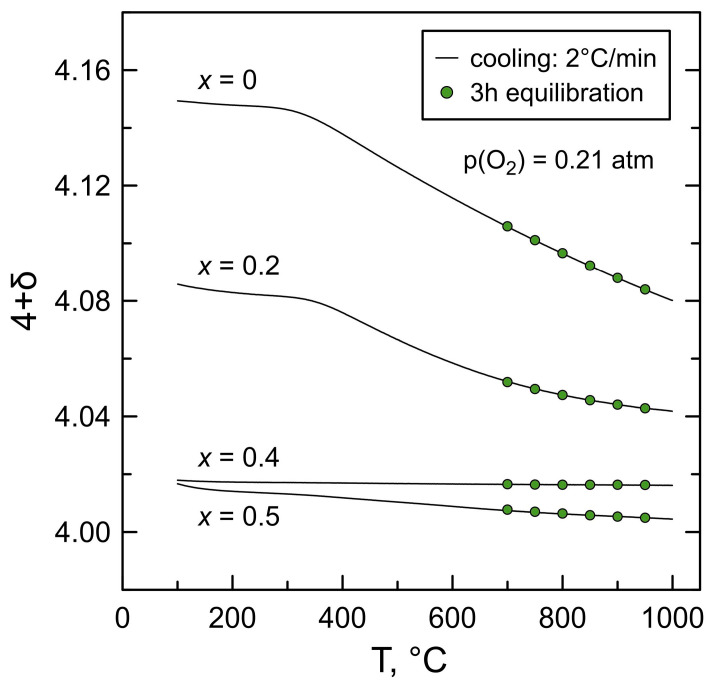
Temperature dependence of oxygen nonstoichiometry of La_2−*x*_Ba*_x_*NiO_4+δ_ ceramics in air. Solid lines correspond to the data obtained on dynamic cooling; circles are the values obtained after 3 h of equilibration at a given temperature.

The results of thermogravimetric studies indicate that La_2−*x*_Ba*_x_*NiO_4+δ_ nickelates with moderate barium contents, *x* ≤ 0.5, remain oxygen-overstoichiometric at atmospheric oxygen pressure and temperatures ≤ 1000 °C (Figure 8).

**Figure 9:**
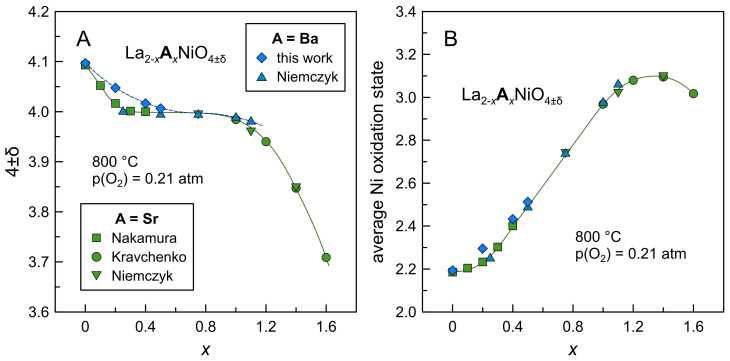
Dependence of oxygen nonstoichiometry (**A**) and average nickel oxidation state (**B**) in Ln_2−*x*_A*_x_*NiO_4±δ_ nickelates on barium and strontium content at 800 °C. Literature data are taken from Nakamura [18], Kravchenko [21], and Niemczyk [23].

For this range of compositions and p(O_2_)-T conditions, the lattice electroneutrality condition is given by (using Kröger-Vink notation):

(3) $$\left[Ba_{\mathrm{La}}^{\prime }]+2[O_{i}^{\prime \prime }]=[Ni_{\mathrm{Ni}}^{•}\right]$$

where $Ba_{\mathrm{La}}^{\prime }$ indicates Ba^2+^ cation in lanthanum sublattice, $O_{i}^{\prime \prime }$ is interstitial oxygen ion, and $Ni_{\mathrm{Ni}}^{•}$ is Ni^3+^ cation (or electron-hole formally residing on nickel cation). The oxygen content is nearly constant in the low-temperature range due to a kinetically frozen oxygen exchange between the oxide lattice and gas phase. Above ~300–350 °C, increasing temperature leads to a decrease in oxygen excess due to the reversible losses of interstitial oxygen from the lattice (Figure 8) and the accompanying reduction of nickel cations:

(4) $$2Ni_{\mathrm{Ni}}^{•}+O_{i}^{\prime \prime }\overset{T↑}{\underset{T↓}{⇄}}2Ni_{\mathrm{Ni}}^{\times }+0.5O_{2}$$

where $Ni_{\mathrm{Ni}}^{\times }$ is a Ni^2+^ cation. According to the electroneutrality condition, Equation (3), increasing concentration of acceptor-type dopant, [Ba^2+^], should be charge-compensated by a decrease in the concentration of interstitial oxygen anions, or increasing concentration of Ni in 3+ oxidation state (generation of electron holes), or both. Calculations showed that substitution by barium is actually charge-compensated by a simultaneous decrease in oxygen excess and formation of Ni^3+^ (Figure 9A,B). Note that electron-hole concentration (*p*) and Ni^3+^ concentration are interrelated with the average oxidation state (*aOS*) of nickel cations as:

(5) $$p=[Ni_{\mathrm{Ni}}^{•}]=aOS-2$$

As defined by the charge compensation mechanism, the extent of oxygen nonstoichiometry changes with temperature is reduced with increasing barium content, and the *x* = 0.4 composition exhibits temperature-independent δ ~ 0.016 in the entire studied temperature range in air. These trends are in very good agreement with the results published earlier for the La_2−*x*_Sr*_x_*NiO_4+δ_ (*x* = 0–0.4) system [18].

La_1.5_Ba_0.5_NiO_4+δ_ expectedly exhibits even lower oxygen nonstoichiometry, δ = 0.005 at 950 °C. However, while one could expect a plateau-like behavior of δ vs. T, similar to the *x* = 0.4 composition, the calculated oxygen content in La_1.5_Ba_0.5_NiO_4+δ_ was found to increase continuously on cooling (Figure 8). This behavior was reproducible for different samples. One should note that the *x* = 0.5 composition is on the border of the single-phase solid solutions formation range obtained in the present work. It is likely that the behavior of these samples on thermal cycling and calculations from the thermogravimetric data are slightly affected by the presence of undetected traces of phase impurities and/or carbonation/hydration of the sample surface during the experiments. Niemczyk et al. [23] reported that La_2−*x*_Ba*_x_*NiO_4±δ_ nickelates exhibit a proton uptake ability which increases with reducing temperature and increasing barium content. Whatever the reason, the apparent changes in oxygen nonstoichiometry in La_1.5_Ba_0.5_NiO_4+δ_ on thermal cycling corresponded to Δδ ~ 0.01 (Figure 8).

Figure 9 summarizes the trends in variations of oxygen nonstoichiometry and average nickel oxidation state in La_2−*x*_Ba*_x_*NiO_4±δ_ and La_2−*x*_Sr*_x_*NiO_4±δ_ series in a wide range of compositions at 800 °C. The results on La_2−*x*_Ba*_x_*NiO_4+δ_ nickelates obtained in the present work are in good agreement with the literature, although the absolute values of δ for Ba-containing compositions are slightly higher compared to literature data. The plot illustrates a gradual transition from oxygen excess at moderate contents of acceptor-type dopant to oxygen stoichiometry in intermediate compositions and eventually to oxygen deficiency in Sr- and Ba-rich nickelates. This is accompanied by a gradual increase in average nickel oxidation state and electron-hole concentration until some limiting value at a given temperature.

### 3.3. Electrical Conductivity

**Figure 10:**
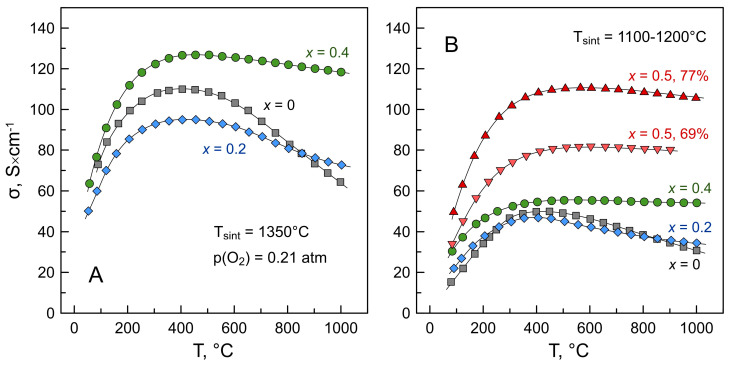
Temperature dependence of electrical conductivity of La_2−*x*_Ba*_x_*NiO_4+δ_ ceramics sintered at 1350 °C (**A**) and 1100–1200 °C (**B**). The fabrication conditions and relative density of all samples are detailed in Table 1.

Figure 10 shows the data on the electrical conductivity of La_2−*x*_Ba*_x_*NiO_4+δ_ (*x* = 0–0.5) ceramics in air. In the case of Ba-rich samples, *x* ≥ 0.6, the obtained results were of inadequate quality and poor reproducibility, apparently due to combined effects of phase impurities, formation/decomposition of carbonates, and reactivity with Pt probes and current collectors; the latter is discussed below in Section 3.6. Therefore, the data on the electrical properties of Ba-rich materials were excluded from the discussion.

La_2_NiO_4+δ_-derived materials are known to be predominantly *p*-type electronic conductors, with oxygen-ionic conductivity ≥ 3 orders of magnitude lower compared to electronic and total conductivity [16]. In the low-temperature range, when the oxygen content in the crystal lattice is fixed (Figure 8), La_2_NiO_4+δ_ and La_2−*x*_Ba*_x_*NiO_4+δ_ ceramics exhibit temperature-activated electrical conductivity. Above ~400–450 °C, the materials show a transition to a pseudo-metallic behavior with conductivity decreasing on heating. This is mainly associated with the loss of oxygen from the crystal lattice accompanied by a decrease in electron-hole concentration as shown by Equation (4). The extent of conductivity variations with temperature (Figure 10) directly correlates with the extent of changes in oxygen nonstoichiometry on thermal cycling (Figure 8): stronger dependencies for *x* = 0–0.2 and very weak ones for nearly oxygen-stoichiometric *x* = 0.4–0.5 compositions.

The results showed that the level of electrical conductivity is strongly affected by the sintering conditions and, consequently, the porosity of the samples. Increasing the volume fraction of pores by 20–30% results in a ~2–2.5 times drop in conductivity (Table 1 and Figure 10). The attempts to approximate the experimental values to zero porosity showed that simple models based on the fraction of porosity only [44] and often used for corrections of experimental conductivity data (e.g., Refs. [45,46]) could not yield adequate results, as was evident from the comparison of “corrected” values for the samples with different porosity.

In general, the electrical conductivity of La_2−*x*_Ba*_x_*NiO_4+δ_ ceramics increases with increasing barium content, in agreement with the changes in average nickel oxidation state (Figure 9) and electron-hole concentration, Equation (5). This is also in accordance with the results reported for the La_2−*x*_Sr*_x_*NiO_4±δ_ system (e.g., Refs. [19,47]). For highly porous La_1.5_Ba_0.5_NiO_4+δ_ ceramics with a relative density of ~70%, electrical conductivity reaches ~80 S/cm at 450–900 °C.

**Figure 11:**
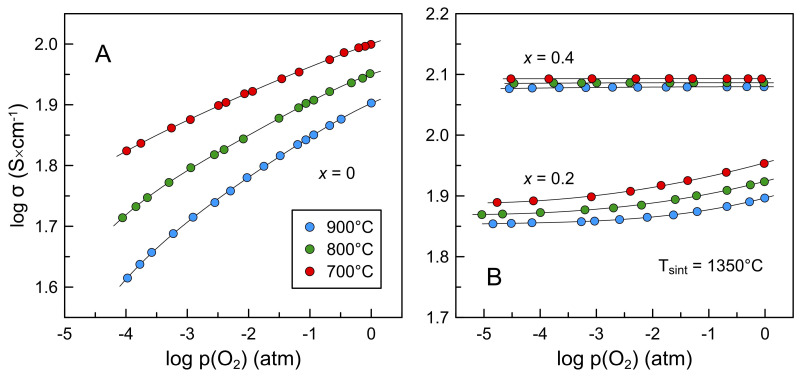
Oxygen partial pressure dependence of electrical conductivity of La_2_NiO_4+δ_ (**A**) and La_2−*x*_Ba*_x_*NiO_4+δ_ (*x* = 0.2 and 0.4) (**B**) ceramics sintered at 1350 °C.

**Figure 12:**
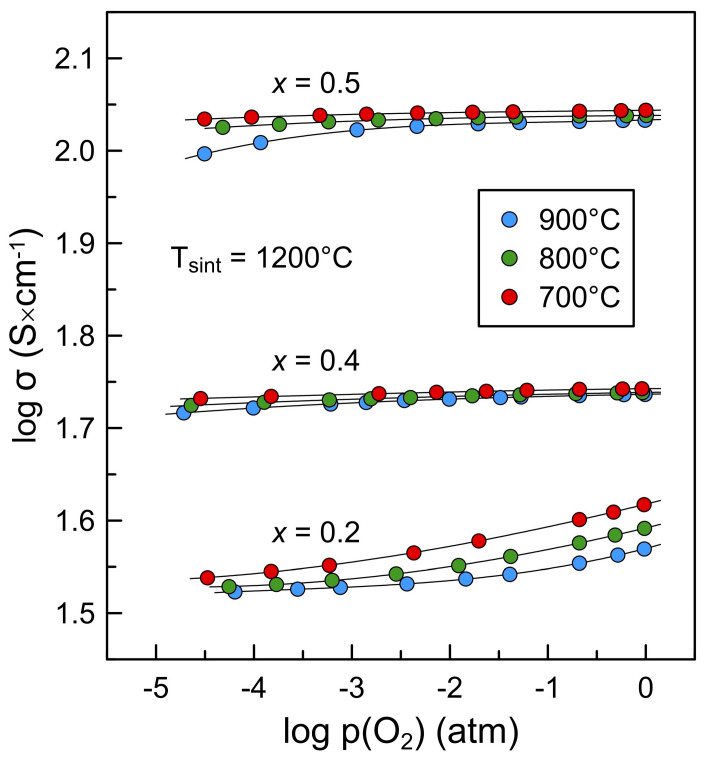
Oxygen partial pressure dependence of electrical conductivity of La_2−*x*_Ba*_x_*NiO_4±δ_ (*x* = 0.2–0.5) ceramics sintered at 1200 °C.

Figure 11 and Figure 12 present isothermal dependencies of electrical conductivity of La_2−*x*_Ba*_x_*NiO_4+δ_ ceramics on oxygen partial pressure at 700–900 °C and p(O_2_) range from 10^−5^ to 1.0 atm. Reducing p(O_2_) results in a decrease in *p*-type electronic conductivity. Once again, this happens due to reversible oxygen release from the oxide lattice and the accompanying reduction in the electron-hole concentration:

(6) $$2Ni_{\mathrm{Ni}}^{•}+O_{i}^{\prime \prime }\overset{pO_{2}↓}{\underset{pO_{2}↑}{⇄}}2Ni_{\mathrm{Ni}}^{\times }+0.5O_{2}$$

As electron-hole concentration is directly interrelated with oxygen nonstoichiometry through the electroneutrality condition, Equation (3), relative changes in electrical conductivity correlate with the extent of oxygen nonstoichiometry variations with p(O_2_) and, consequently, barium content. Undoped La_2_NiO_4+δ_ exhibits variable oxygen nonstoichiometry in the studied p(O_2_) range [18,48] and, therefore, a strong p(O_2_)-dependence of electrical conductivity. On the contrary, nearly oxygen-stoichiometric La_1.6_Ba_0.4_NiO_4+δ_ is expected to possess a weak dependence of δ on oxygen partial pressure and shows nearly p(O_2_)-independent electrical conductivity. La_1.8_Ba_0.2_NiO_4+δ_ demonstrates an intermediate behavior, with conductivity tending to a plateau at reduced oxygen pressures. The obtained results resemble well the corresponding data reported for the La_2−*x*_Sr*_x_*NiO_4+δ_ (*x* = 0–0.4) system [19]. The behavior of La_1.5_Ba_0.5_NiO_4+δ_ ceramics at 700–800 °C was found to be similar to that of the *x* = 0.4 composition (Figure 12). At the same time, the slope of *log σ–log p(O_2_)* dependence tends to increase with reducing p(O_2_) at 900 °C, thus suggesting a transition to oxygen deficiency regime at reduced oxygen partial pressure.

Post-mortem XRD analysis of the samples after electrical conductivity measurements confirmed the redox stability of La_2−*x*_Ba*_x_*NiO_4+δ_ (*x* ≤ 0.5) nickelates in the studied range of T-p(O_2_) conditions, with no evidence of reductive phase decomposition in the XRD patterns.

### 3.4. Oxygen Permeability and Ionic Transport

**Figure 13:**
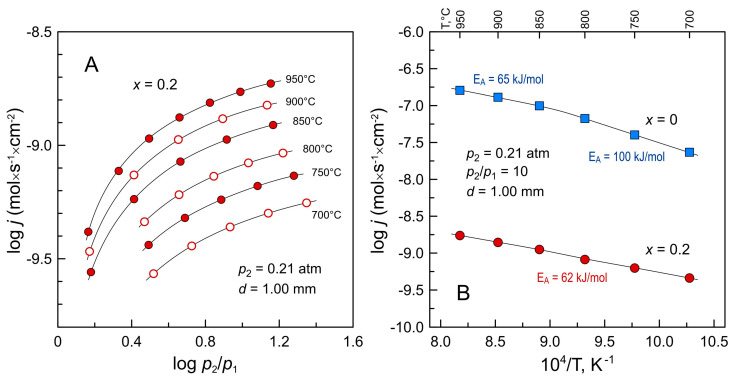
(**A**) Dependence of oxygen permeation fluxes through La_1.8_Ba_0.2_NiO_4+δ_ ceramic membrane on oxygen partial pressure gradient at 700–950 °C; (**B**) Temperature dependence of oxygen permeation fluxes through La_2−*x*_Ba*_x_*NiO_4+δ_ ceramic membranes under fixed p(O_2_) gradient. *d* is membrane thickness; *p*_2_ and *p*_1_ are oxygen partial pressures at the membrane feed and permeate side, respectively. The membranes were sintered at 1350 °C.

Oxygen permeability of La_2−*x*_Ba*_x_*NiO_4+δ_ (*x* = 0 and 0.2) nickelates, the only two materials obtained in the form of dense gas-tight ceramics, was studied as a function of temperature and oxygen partial pressure gradient across the membrane. The results are presented in Figure 13. It was found that the substitution of 10 at.% of lanthanum by barium results in ~2 orders of magnitude drop in oxygen permeation fluxes through ceramic membranes at 700–950 °C. Oxygen-ionic transport in RP-type Ln_2_NiO_4+δ_-based nickelates is considered to occur by diffusion of interstitial oxygen ions along the rock-salt-type layers via the so-called interstitialcy mechanism [16,49]. Thus, the decline in oxygen-ionic transport in Ba-substituted nickelate should be assigned mainly to the decrease in the concentration of mobile interstitial oxygen ions (Figure 8). For comparison, an even more substantial drop in oxygen permeation fluxes, nearly three orders of magnitude, was reported for calcium-substituted La_1.7_Ca_0.3_NiO_3+δ_ membranes compared to undoped La_2_NiO_4+δ_ [50]. The calculated activation energy E_A_ of oxygen permeation flux through La_1.8_Ba_0.2_NiO_4+δ_ ceramic membranes at 700–950 °C is similar to that for undoped La_2_NiO_4+δ_ nickelate membrane in the high-temperature range (Figure 13B). This suggests that the mechanism of oxygen-ion diffusion remains unchanged and supports the conclusion that the drop in permeation flux with barium doping is due to the decrease in the concentration of mobile ionic charge carriers. Note that an increase in activation energy of oxygen permeability of La_2_NiO_4+δ_ ceramics at temperatures below 850 °C occurs due to the increasing limiting effect of surface exchange kinetics (e.g., Ref. [51]). On the contrary, the overall oxygen transport across the barium-substituted La_1.8_Ba_0.2_NiO_4+δ_ membrane is limited mainly by bulk diffusion, and the activation energy remains the same in the studied temperature range.

The values of partial oxygen-ionic conductivity σ_O_ of a mixed ionic-electronic conductor (MIEC) can be roughly estimated from the data on oxygen permeability and total electrical conductivity using the Wagner equation for the steady-state oxygen permeation flux *j* through the MIEC membrane bulk [52]:

(7) $$j=\frac{\mathrm{RT}}{16F^{2}d}\int_{p_{1}}^{p_{2}}\frac{\sigma_{O}\sigma_{e}}{\sigma_{O}+\sigma_{e}}\partial \ln p(O_{2})$$

where *d* is the membrane thickness, *p*_2_ and *p*_1_ are oxygen partial pressures at the membrane feed and permeate sides, respectively, and σ_e_ is the partial electronic conductivity. Such estimations ignore the limiting effect of the oxygen exchange rates at the membrane surfaces and may yield somewhat underestimated σ_O_ values but are suitable for determining the order of magnitude of ionic conductivity. The calculations from the oxygen permeation data under minimum p(O_2_) gradients (*p*_1_ ≥ 0.08 atm) and total conductivity in air showed that oxygen-ionic conductivity decreased from ~0.11 S/cm in undoped La_2_NiO_4+δ_ to ~1.2×10^−3^ S/cm in La_1.8_Ba_0.2_NiO_4+δ_ at 900 °C. Note that the value for the parent nickelate is in good agreement with the literature data [16].

### 3.5. Thermal Expansion

**Figure 14:**
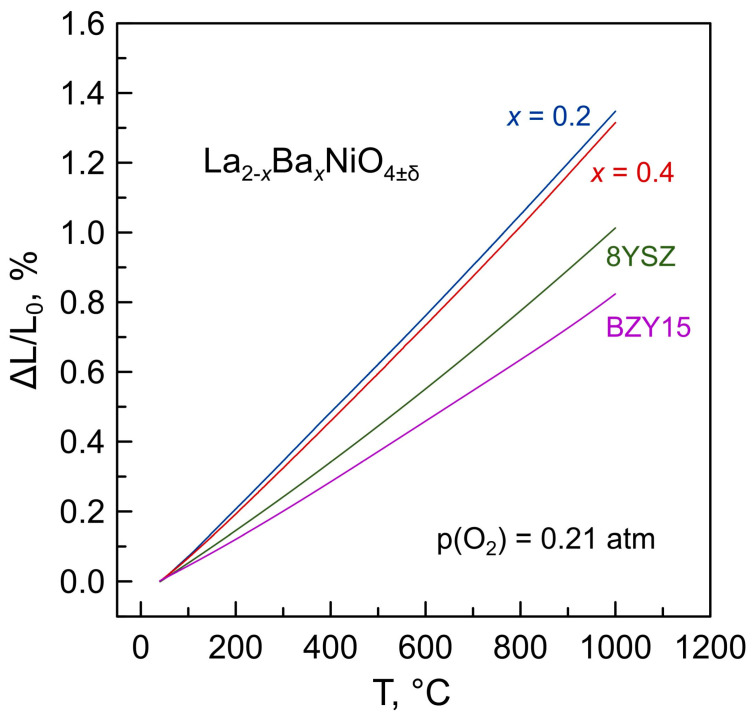
Dilatometric curves of La_2−*x*_Ba_*x*_NiO_4+δ_ and selected solid electrolyte ceramics in air.

Dimensional changes of La_2−*x*_Ba*_x_*NiO_4+δ_ (*x* = 0–0.5) ceramics on thermal cycling in air between room temperature and 1000 °C were studied by dilatometry. Selected dilatometric curves in comparison with the data on 8YSZ and BZY15 solid electrolytes are shown in Figure 14. La_2−*x*_Ba*_x_*NiO_4+δ_ ceramics exhibit smooth, nearly linear thermal expansion in air. The average thermal expansion coefficients (TECs) in the studied temperature range vary in a narrow range, 13.8–14.3 ppm/K (Table 2), and tend to decrease slightly with increasing barium content. The latter can be attributed to decreasing chemical contribution to thermochemical expansion. Chemical expansion originates from the variations in oxygen nonstoichiometry δ and, consequently, the average ionic size of nickel cations in the crystal lattice on thermal cycling [42] and should vanish with increasing barium content, as follows from the data on oxygen nonstoichiometry (Figure 8).

The average TECs of La_2−*x*_Ba*_x_*NiO_4+δ_ ceramics exceed that of traditional 8YSZ solid electrolytes (Table 2). The difference is still acceptable from the point of view of thermomechanical compatibility, as can be concluded from the multiple literature reports on the electrochemical characterization of La_2_NiO_4+δ_-based electrodes applied onto 8YSZ. On the other hand, a significant mismatch between the thermal expansion coefficients of La_2−*x*_Ba*_x_*NiO_4+δ_ nickelates and BZY15 solid electrolyte should constitute a critical issue for the thermomechanical stability of the electrode/electrolyte interface. This can be solved by fabricating composite (La,Ba)_2_NiO_4+δ_ + BZY15 electrodes to adjust the TEC values of electrode layers.

### 3.6. High-Temperature Chemical Compatibility with Other Materials

The results of chemical compatibility tests are summarized in Table 3. XRD analysis of pelletized mixtures of La_2−*x*_Ba*_x_*NiO_4+δ_ and 8YSZ powders revealed the formation of a poorly conducting La_2_Zr_2_O_7_ pyrochlore-type phase after annealing at 700 °C for 72 h. The high reactivity between La_2_NiO_4−δ_ and 8YSZ at 900 °C was noted earlier in [53]. Thus, undesirable chemical reactivity even at temperatures as low as 700 °C is problematic for the practical application of the studied nickelates in direct contact with yttria-stabilized zirconia.

**Figure 15:**
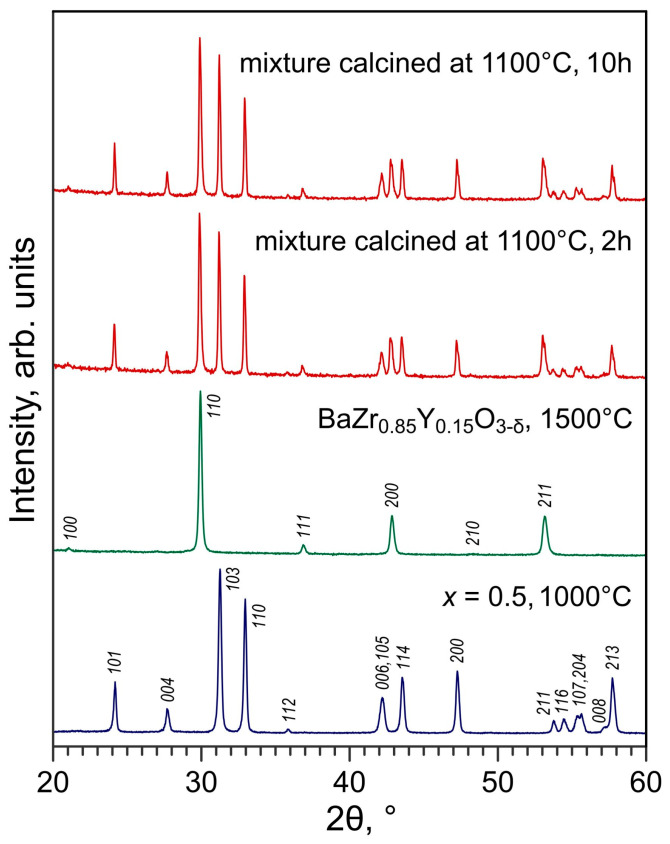
XRD patterns of powdered La_1.5_Ba_0.5_NiO_4+δ_ + BZY15 mixture (50:50 wt.%), annealed at 1100 °C for 2 and 10 h, and individual materials before mixing. XRD pattern of tetragonal La_1.5_Ba_0.5_NiO_4+δ_ is indexed in space group *I*4/*mmm*; XRD pattern of cubic BaZr_0.85_Y_0.15_O_3−δ_ (BZY15) is indexed in space group *Pm*-3*m* according to ICDD PDF 04-016-4803.

Good compatibility with no evidence of segregation of reactivity products was found for La_2−*x*_Ba*_x_*NiO_4±δ_ + BZY15 pairs under identical annealing conditions. The traces of barium carbonate observed in the XRD patterns originate from commercial BZY15 powder (used in the as-delivered state) and can be eliminated by calcination at ≥1300 °C. Thus, BZY15 ceramics with reported oxygen-ion transference numbers of 0.7–0.9 in the temperature range of 500–600 °C in wet O_2_ [54] seems a reasonable alternative as a solid electrolyte for electrocatalytic tests of La_2−*x*_Ba*_x_*NiO_4±δ_ nickelates. In order to evaluate the compatibility of nickelates with barium zirconate-based ceramics at higher temperatures corresponding to anticipated electrode fabrication conditions, the mixture of La_1.5_Ba_0.5_NiO_4+δ_ and BZY15 powders was annealed at 1100 °C for 2 and 10 h and then examined by XRD. For these tests, BZY15 compacts were sintered at 1500 °C and then crushed into powder, while La_1.5_Ba_0.5_NiO_4+δ_ powder was used in the as-synthesized form (i.e., after calcinations at 1000 °C in air). No evidence of reactivity or formation of secondary phases could be observed in the XRD patterns of calcined mixtures (Figure 15) confirming good chemical compatibility between the materials at temperatures at least up to 1100 °C.

**Figure 16:**
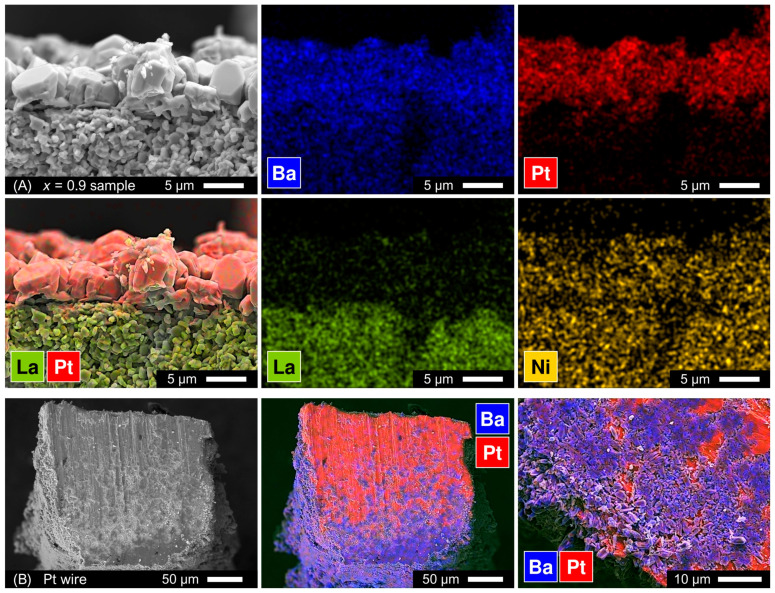
SEM images and corresponding EDS elemental mapping results for the samples after electrical conductivity measurements at T ≤ 1000 °C: (**A**) Fractured cross-section of La_1.1_Ba_0.9_NiO_4±δ_ ceramic samples near the surface (distance from the Pt wire probe: ~3 mm); (**B**) Cut cross-section of Pt wire (potential probe) after contact with the La_1.1_Ba_0.9_NiO_4±δ_ ceramics during the measurements.

As mentioned above in Section 3.3, the results of electrical conductivity measurements of Ba-rich La_2−*x*_Ba*_x_*NiO_4±δ_ samples (*x* ≥ 0.6) were poorly reproducible and unreliable. Selected samples after electrical studies were inspected by SEM/EDS; an example of the analysis is presented in Figure 16A. Microscopic studies revealed the formation of a surface layer of agglomerated oxide particles (up to 8 µm in diameter) rich in Ba, Ni, and Pt, and free of La. The layer of such agglomerates formed not only in the vicinity of Pt wire probes but on the entire surface of bar-shaped samples indicating a strong surface diffusion of platinum. Inspection of the Pt wire probe that was in contact with the ceramic sample during the measurement also revealed a significant diffusion of barium oxide inside the wire bulk (Figure 16B), although no nickel could be detected in this case. Massive reactivity was also detected at the interface between ceramic samples and porous Pt electrodes (applied at the end-face surface of the bar-shaped samples before measurements).

Several compounds and a wide range of solid solutions were reported to exist in the BaO-Pt(PtO_2_) system in air [55,56]. Furthermore, Ba (in the form of BaO, BaCO_3_ or Ba(OH)_2_) was reported to react readily with platinum at 600–700 °C in air to form BaPtO_3_ [57,58]. Thus, one may conclude that the reactivity between Ba-rich La_2−*x*_Ba*_x_*NiO_4±δ_ samples and Pt components is caused by the presence of Ba-based phase impurities (BaO, BaCO_3_, and Ba-Ni-O phases) in the samples. No evidence of surface reactivity could be detected for the La_2−*x*_Ba*_x_*NiO_4+δ_ samples with moderate barium contents, *x* ≤ 0.5, after electrical conductivity measurements for 1–2 weeks at temperatures up to 1000 °C. Another noteworthy observation is that annealing of as-sintered polished La_1.2_Ba_0.8_NiO_4±δ_ ceramic sample for ~500 h at 700 °C in air did not promote any surface segregation, and the surface and bulk morphology remained identical. This means that the formation of Ba-Ni-Pt-O layers at the surface of the sample during the electrical measurements is provoked by surface Pt diffusion.

The reactivity between La_1.2_Ba_0.8_NiO_4±δ_ and metallic platinum with the formation of BaPtO_3_ was confirmed by XRD analysis of the pelletized mixture of powders annealed at 700 °C for 72 h (Table 3). The reactivity is likely to be promoted by the melting of secondary phases, as suggested by the formation of hexagonal Ba-Pt-O phases observed at 1300 °C (Figure 6) and also after cooling during variable-temperature XRD studies (Figure S5). Gold showed much better chemical compatibility with Ba-rich samples in annealing tests (Table 3); gold wires can be used as an alternative to Pt probes for electrical studies of such ceramic materials.

## 4. Conclusions

Ceramic materials of the La_2−*x*_Ba*_x_*NiO_4±δ_ (*x* = 0–1.1) series were prepared by glycine-nitrate combustion route followed by calcinations at 800–1100 °C and sintering at 1100–1350 °C in air or oxygen atmosphere. The range of formation of single-phase solid solutions under applied synthetic conditions was found to be limited to *x* = 0.5. For Ba-rich composition (*x* ≥ 0.6), La_2_NiO_4_-based solid solutions co-existed with secondary phases: BaO (or BaCO_3_), NiO, and various phases of the Ba-Ni-O system. Acceptor-type substitution of lanthanum by barium is charge-compensated by decreasing the concentration of interstitial oxygen ions, from δ ~ 0.1 for *x* = 0 to δ ~ 0.01 for *x* = 0.4–0.5 at 800 °C in air, and generation of electron-holes (i.e., formation of Ni^3+^). This leads to an increase in *p*-type electronic conductivity and a decline in oxygen-ionic transport. Electrical conductivity of highly porous La_1.5_Ba_0.5_NiO_4+δ_ ceramics with a relative density of ~70% reaches ~80 S/cm at 450–900 °C. The average TECs of La_2−*x*_Ba*_x_*NiO_4+δ_ (*x* = 0–0.5) ceramics vary in a narrow range of 13.8–14.3 ppm/K at 25–1000 °C in air. La_2−*x*_Ba*_x_*NiO_4±δ_ nickelates show reactivity with YSZ solid electrolyte at 700 °C, with the formation of insulating La_2_Zr_2_O_7_ phase, but good chemical compatibility with BZY15 electrolyte. Ba-rich compositions exhibited strong reactivity with Pt caused by the presence of Ba-based phase impurities. La_1.5_Ba_0.5_NiO_4+δ_ ceramics appear to be best-suited for potential application in the electrochemical elimination of NO*_x_* exhibiting sufficiently high electrical conductivity, phase stability in air up to at least 1200 °C, and good chemical compatibility with BaZr_0.85_Y_0.15_O_3−δ_ solid electrolyte at temperatures up to at least 1100 °C, which enables the fabrication of porous electrode layers without undesirable reactivity at these temperatures.

## Figures and Tables

**Table 1:** Sintering conditions, density, and electrical conductivity of La_2−*x*_Ba*_x_*NiO_4+δ_ ceramics.

*x*	Sintering Conditions	Density, g/cm^3^	Relative Density, %	Electrical Conductivity σ, S/cm	
				450 °C	800 °C
0	1350 °C, air	6.84	96.8	109.7	83.7
	1100 °C, air	5.52	78.0	49.9	38.5
0.2	1350 °C, air	6.49	92.2	95.0	81.1
	1200 °C, air	5.02	71.2	46.5	37.7
0.4	1350 °C, air	6.53	93.4	126.8	122.0
	1200 °C, air	4.46	63.7	55.1	54.6
0.5	1200 °C, air	5.33	76.5	110.0	109.1
	1200 °C, O_2_	4.78	68.6	79.8	80.2

**Table 2:** Average thermal expansion coefficients ($\bar{\alpha }$) calculated from the dilatometric data in air.

Composition	T, °C	($\bar{\alpha }$ × 10^6^) ± 0.1, K^−1^
La_2_NiO_4+δ_	30–1000	14.3
La_1.8_Ba_0.2_NiO_4+δ_	30–1000	14.1
La_1.6_Ba_0.4_NiO_4+δ_	30–1000	13.8
La_1.5_Ba_0.5_NiO_4+δ_	30–1000	14.0
8YSZ, (ZrO_2_)_0.92_(Y_2_O_3_)_0.08_	30–1100	10.5
BZY15, BaZr_0.85_Y_0.15_O_3−δ_	30–1100	8.5

**Table 3:** Chemical compatibility between La_2−*x*_Ba*_x_*NiO_4±δ_ and other materials of solid oxide cells.

Tested Pairs of Materials	XRD ^1^	SEM/EDS ^2^
La_2−*x*_Ba*_x_*NiO_4+δ_ + 8YSZ (*x* = 0.4, 0.5)	traces of La_2_Zr_2_O_7_	–
La_2−*x*_Ba*_x_*NiO_4±δ_ + BZY15 (*x* = 0.4, 0.5, 0.8)	no reactivity, traces of BaCO_3_	–
La_1.2_Ba_0.8_NiO_4±δ_ + Pt	BaPtO_3_^3^	Ba-Ni-Pt-O phases at the surface
La_1.2_Ba_0.8_NiO_4±δ_ + Au	no reactivity	–

^1^ Mixtures of powders were pressed in pellets and annealed for 72 h at 700 °C in air; ^2^ Analysis of interfaces after electrical conductivity measurements; ^3^ ICDD PDF 00-034-0850.

## Data Availability

Data are contained within the article and/or available from the corresponding author upon reasonable request.

## Supplementary Materials

The following supporting information can be downloaded at: https://www.mdpi.com/article/10.3390/ma16041755/s1, Figure S1: XRD data—Example of evolution of the phase composition during synthesis; Figure S2: example of XRD pattern of a sample after reduction in the 10% H_2_-N_2_ atmosphere; Figure S3: XRD patterns of ceramics sintered at 1350–1450 °C; Figure S4: SEM/EDS data for La_1.6_Ba_0.4_NiO_4±δ_ ceramics sintered at 1350 °C; Figure S5: room-temperature XRD pattern of La_1.2_Ba_0.8_NiO_4±δ_ ceramic sample after variable-temperature XRD studies at 25–1300 °C.
