# Supplementary material for: Characterization of Ruddlesden-Popper La2−xBaxNiO4±δ Nickelates as Potential Electrocatalysts for Solid Oxide Cells
Source: Materials (Basel). 2023 Feb 20;16(4):1755. doi: 10.3390/ma16041755 (PMC9965424; doi:10.3390/ma16041755)
Supplement: Supplementary file 1 [file materials-16-01755-s001.zip › materials-2193578-supplementary.pdf]

Supplementary Materials

Characterization of Ruddlesden-Popper $La_{2-x}Ba_xNiO_{4\pm\delta}$ Nickelates as Potential Electrocatalysts for Solid Oxide Cells

Kiryl Zakharchuk *, Andrei Kovalevsky and Aleksey Yaremchenko *

CICECO—Aveiro Institute of Materials, Department of Materials and Ceramic Engineering, University of Aveiro, 3810-193 Aveiro, Portugal; akavaleuski@ua.pt

* Correspondence: k.zakharchuk@ua.pt (K.Z.); ayaremchenko@ua.pt (A.Y.)

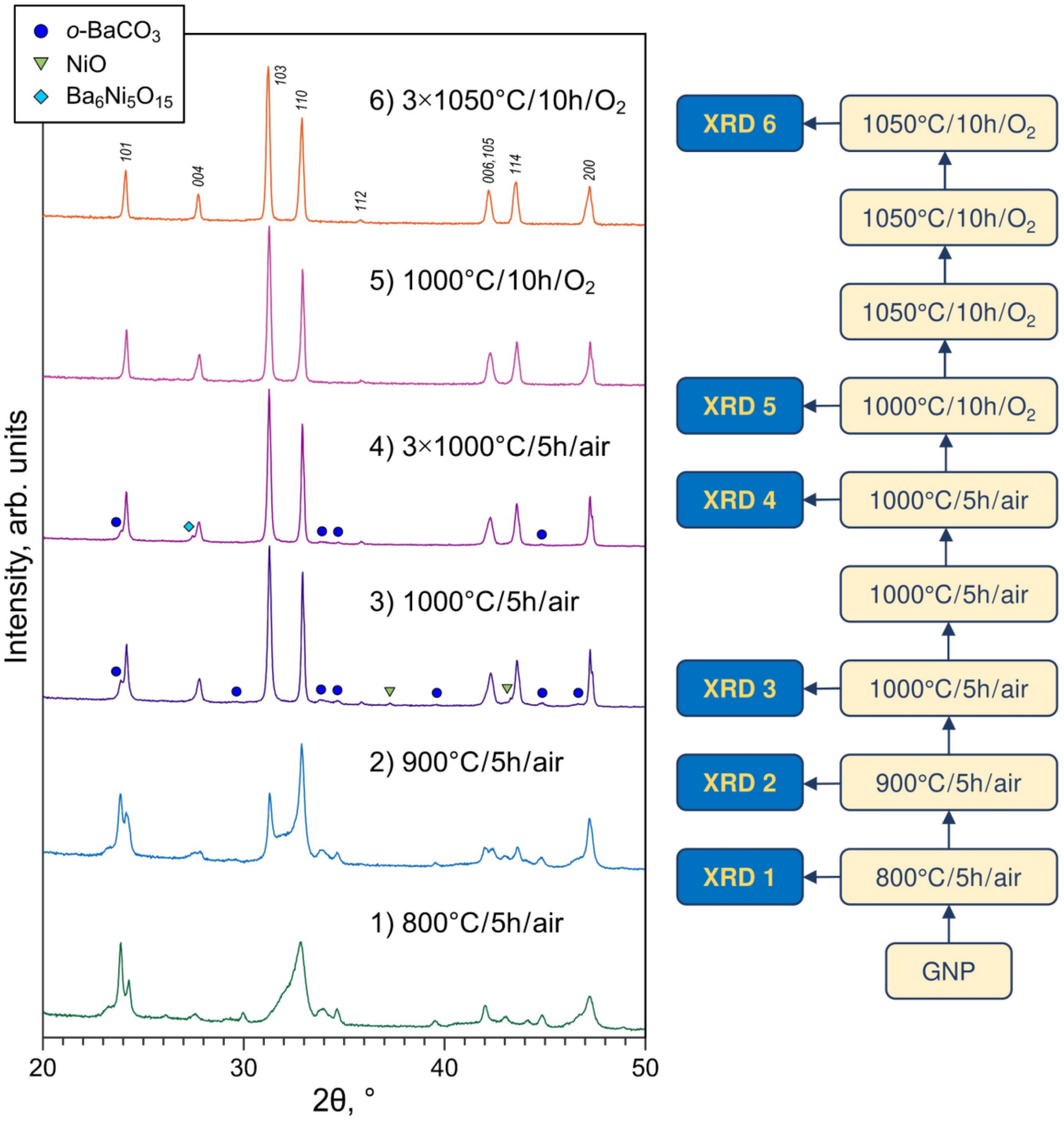

Figure S1. XRD patterns (left) showing the evolution of phase composition of $La_{1.6}Ba_{0.4}NiO_{4\pm\delta}$ ceramic powder in the course of step-wise calcinations with intermediate regrindings. The scheme (right) explains the sequence and conditions of the calcination steps. The reflections of the target phase are indexed in the *I*4/*mmm* space group. The secondary phases are marked according to ICDD PDF 04-015-3214 (orthorhombic α-$BaCO_3$), 01-089-7390 (cubic NiO), and 04-009-3992 (rhombohedral $Ba_6Ni_5O_{15}$).

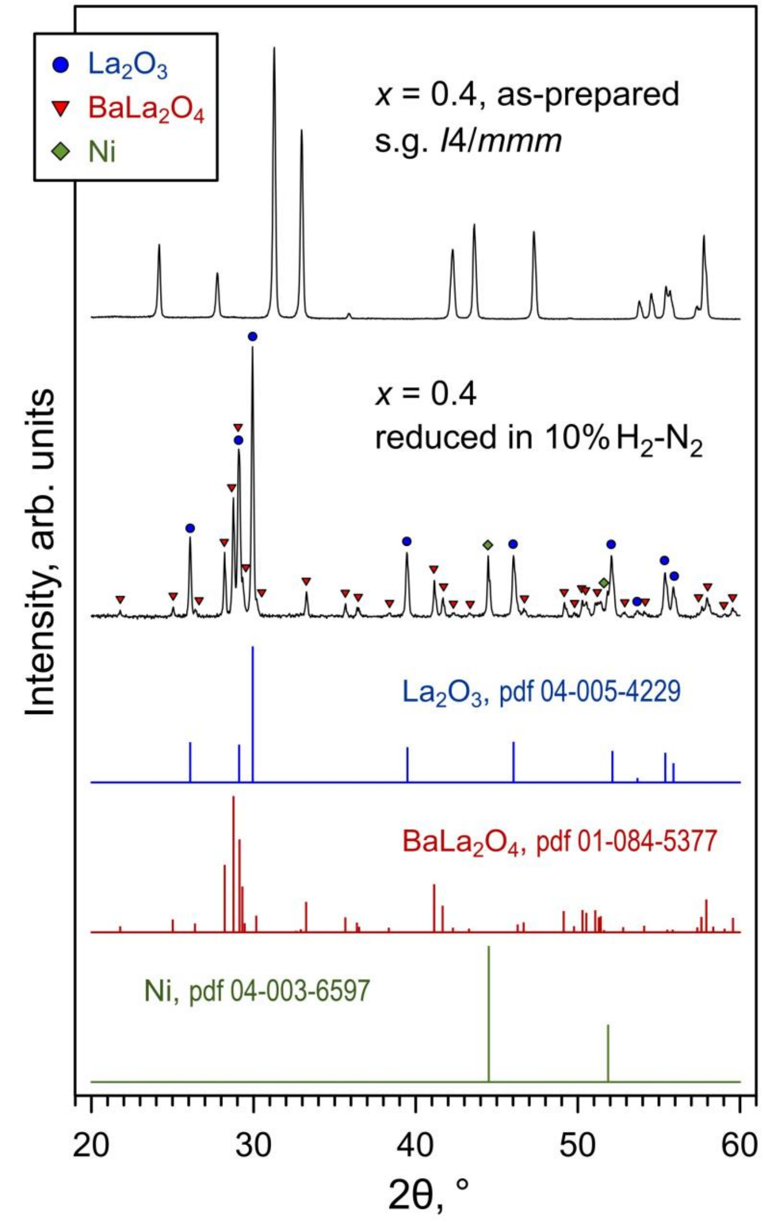

Figure S2. XRD patterns of $La_{1.6}Ba_{0.4}NiO_{4\pm\delta}$ ceramics: as-prepared and after in-situ reduction in 10% H_2-N_2 flow in thermogravimetric equipment.

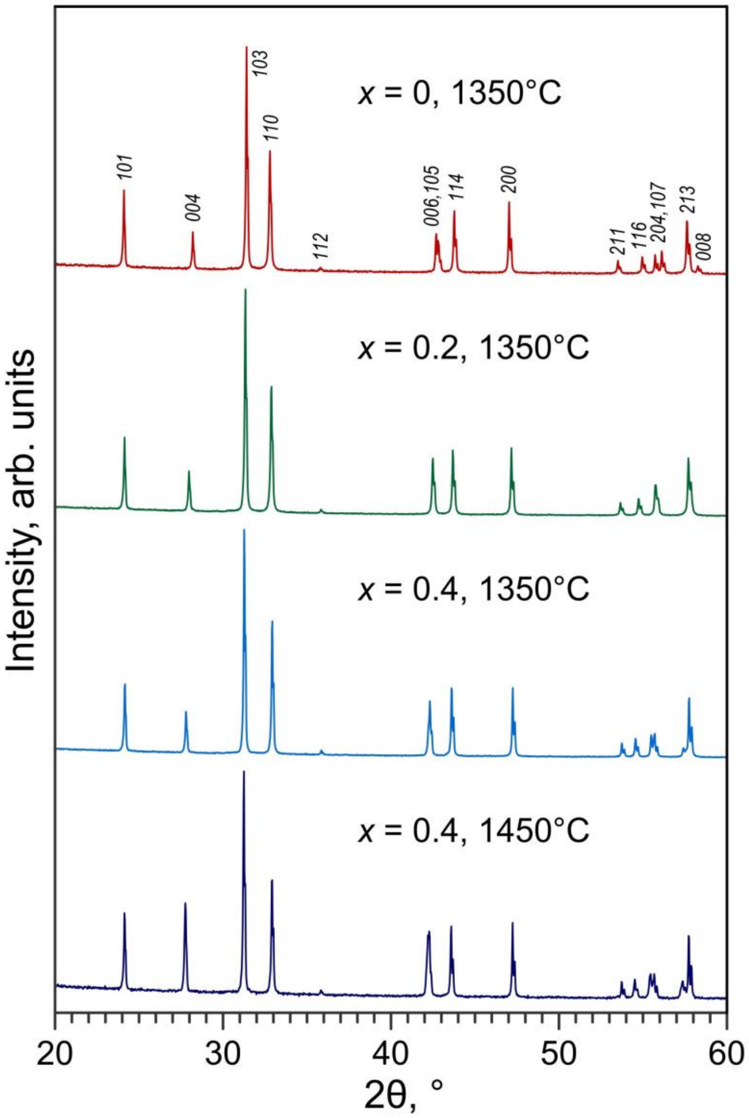

Figure S3. XRD patterns of $La_{2-x}Ba_xNiO_{4+\delta}$ (x = 0-0.4) ceramics sintered at 1350-1450 °C. The reflections are indexed in the tetragonal *I*4/*mmm* space group.

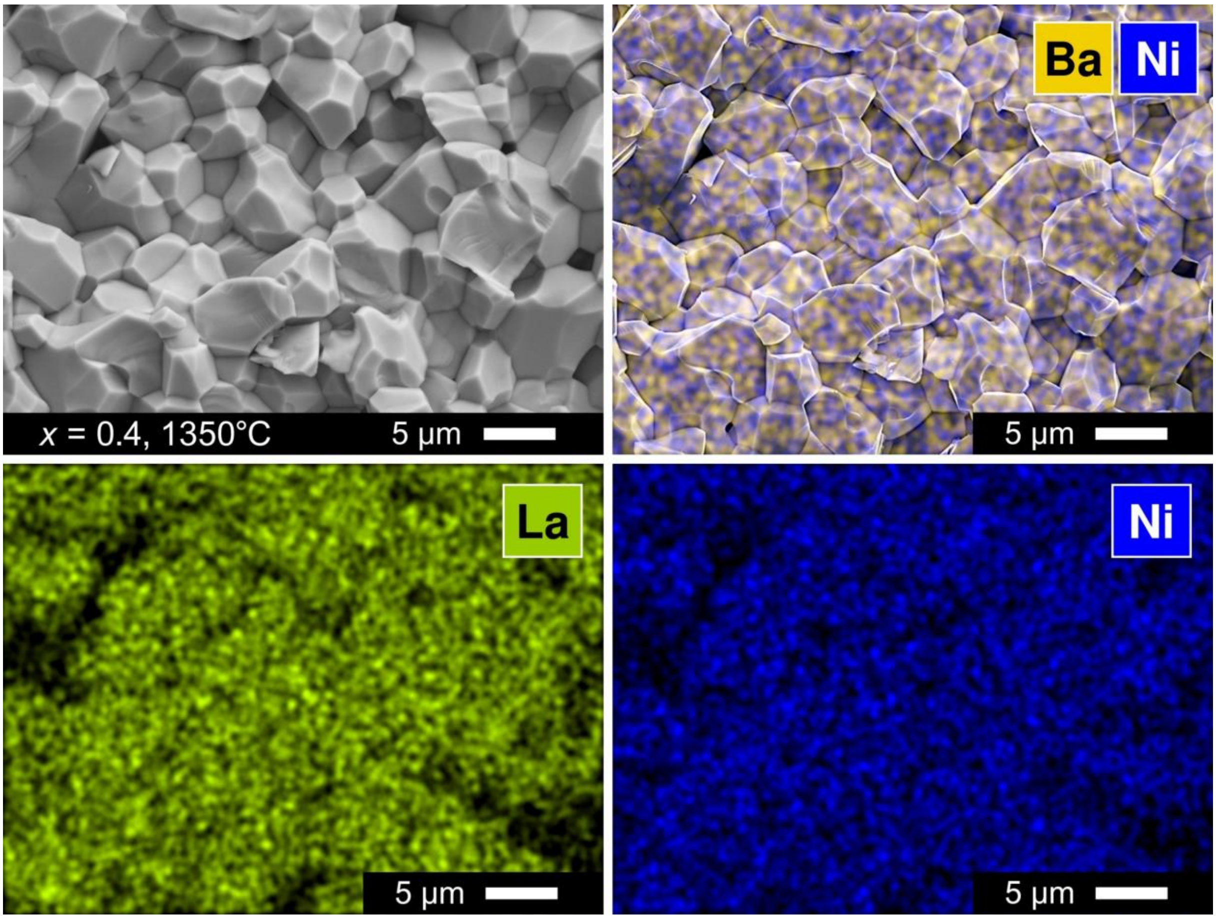

Figure S4. SEM micrograph of the fractured cross-section of $La_{1.6}Ba_{0.4}NiO_{4\pm\delta}$ ceramics sintered at 1350 °C and corresponding EDS elemental mapping results.

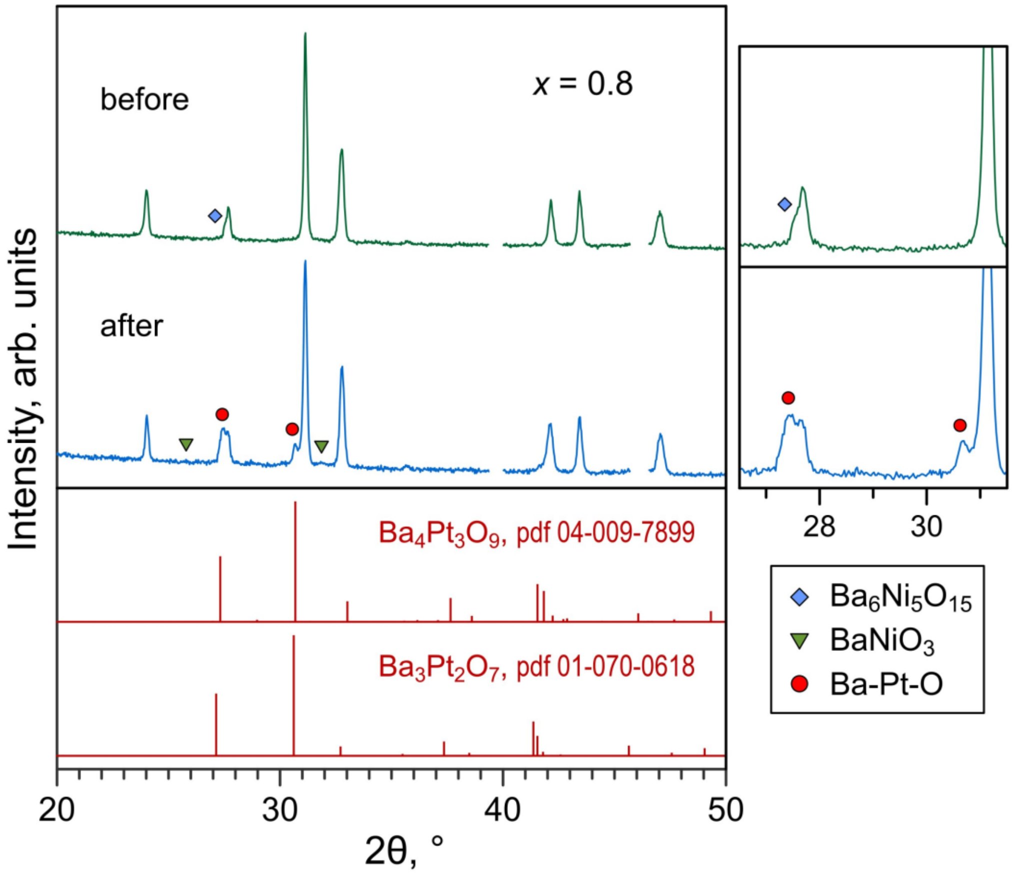

Figure S5. Room-temperature XRD patterns of $La_{1.2}Ba_{0.8}NiO_{4\pm\delta}$ ceramic sample: as-prepared (T_{sint} = 1100 °C) and after variable-temperature XRD studies at 25-1300 °C. Reflections of Pt foil support are cut out. The insets on the right demonstrate enlarged fragments of the diffractograms. The Ba-Ni-O secondary phases are marked according to ICDD PDF 04-009-3992 (rhombohedral $Ba_6Ni_5O_{15}$) and 04-007-8462 (hexagonal $BaNiO_3$).
